# Immunotherapy plus targeted therapy for recurrent or metastatic head and neck squamous cell carcinoma: an updated meta-analysis and subgroup analysis by target mechanism

**DOI:** 10.3389/fonc.2026.1892431

**Published:** 2026-08-03

**Authors:** Wen Sun, Yanlin Wang, Hui Dong, Dianwei Liu

**Affiliations:** 1 Department of Otolaryngology, Yantaishan Hospital, Yantai, Shandong, China; 2 Department of Ophthalmology, Yantaishan Hospital, Yantai, Shandong, China; 3 Department of Oral and Maxillofacial Surgery Ward, Stomatological Hospital of Shandong Medical and Pharmaceutical University / Yantai Stomatological Hospital, Yantai, Shandong, China

**Keywords:** head and neck squamous cell carcinoma, immunotherapy, meta-analysis, recurrent/metastatic, subgroup analysis, targeted therapy

## Abstract

**Background:**

Head and neck squamous cell carcinoma (HNSCC) is an aggressive malignancy with dismal outcomes for patients with recurrent or metastatic (R/M) disease. Although immune checkpoint inhibitors (ICIs) have reshaped the treatment paradigm for R/M HNSCC, the efficacy of monotherapy remains limited. This meta-analysis was performed to comprehensively evaluate the efficacy and safety of immunotherapy combined with targeted therapy for R/M HNSCC and to clarify sources of between-study heterogeneity.

**Methods:**

We systematically searched PubMed, Embase, the Cochrane Library, and Web of Science from inception to May 1, 2026. Eligible studies enrolled patients with histologically confirmed R/M HNSCC receiving immunotherapy combined with targeted therapy and reported predefined efficacy or safety endpoints. Pooled analyses were conducted using Review Manager 5.4 to estimate rates of complete response (CR), partial response (PR), stable disease (SD), objective response rate (ORR), disease control rate (DCR), median progression-free survival (PFS), median overall survival (OS), and grade ≥3 treatment-related adverse events (TRAEs). Subgroup analyses stratified by targeted therapy mechanism were performed to explore heterogeneity.

**Results:**

A total of 22 studies comprising 1118 patients were included. Pooled estimates were as follows: CR rate 0.08 (95% CI: 0.05–0.10), PR rate 0.35 (95% CI: 0.27–0.42), ORR 0.41 (95% CI: 0.32–0.49), SD rate 0.26 (95% CI: 0.22–0.30), DCR 0.75 (95% CI: 0.66–0.83), median PFS 6.9 months, median OS 14.8 months, and grade ≥3 TRAEs rate 0.37 (95% CI: 0.24–0.50). Significant heterogeneity was detected across most endpoints and could not be alleviated by sensitivity analysis. Subgroup analyses demonstrated that anti-EGFR combinations achieved the highest ORR and DCR, whereas novel targeted combinations exhibited a more favorable safety profile.

**Conclusions:**

Immunotherapy combined with targeted therapy yields promising efficacy and manageable toxicity in patients with R/M HNSCC. The type of targeted agent represents a major source of heterogeneity. Anti-EGFR plus immunotherapy regimens show robust antitumor activity, while novel targeted combinations exhibit better tolerability. These findings support mechanism-based individualized combination strategies for R/M HNSCC in clinical practice.

**Systematic Review Registration:**

https://www.crd.york.ac.uk/PROSPERO/, identifier CRD420251050561.

## Introduction

Head and neck squamous cell carcinoma (HNSCC) ranks as the seventh most prevalent malignancy worldwide, with over 940,000 new cases and more than 480,000 deaths annually, and more than 60% of patients present with locally advanced disease at diagnosis (1). Despite multidisciplinary treatments including surgery, radiotherapy and chemotherapy, approximately 40%–60% of patients eventually develop recurrence or distant metastasis, leading to dismal prognosis with median overall survival (OS) of less than one year for recurrent or metastatic (R/M) HNSCC (1, 2). In the past decade, immune checkpoint inhibitors (ICIs) represented by programmed cell death protein 1 (PD-1) inhibitors have revolutionized the therapeutic landscape of R/M HNSCC (3, 4). Pembrolizumab and nivolumab have been approved as first-line or salvage therapies for R/M HNSCC based on the results of KEYNOTE-048 and CheckMate 141 studies, respectively (3, 4). However, the objective response rate (ORR) of PD-1 inhibitor monotherapy is only 13.3%–19%, and most patients eventually develop primary or secondary resistance (5, 6). Therefore, exploring effective combination strategies to enhance antitumor activity and overcome resistance has become an urgent clinical need.

Epidermal growth factor receptor (EGFR) is overexpressed in more than 80% of HNSCC and is closely associated with tumor proliferation, invasion, metastasis and poor prognosis (7, 8). Cetuximab, an anti-EGFR monoclonal antibody, was the first targeted drug approved for R/M HNSCC and has been used in combination with chemotherapy for decades (9). Preclinical studies have confirmed that EGFR inhibitors can upregulate PD-L1 expression in tumor microenvironment, remodel immune cell infiltration, and synergize with PD-1 inhibitors to enhance antitumor effects (10, 11). On this basis, immunotherapy combined with targeted therapy has become a promising strategy for R/M HNSCC. In recent years, a variety of combination regimens have been explored in clinical studies, including PD-1 inhibitors plus cetuximab (12), PD-1 inhibitors plus EGFR/MET bispecific antibodies (13), PD-1 inhibitors plus anti-angiogenic tyrosine kinase inhibitors (TKIs) (14), and PD-1 inhibitors plus CD47 inhibitors (15).

Although some clinical studies have reported encouraging efficacy and safety data of immunotherapy combined with targeted therapy for R/M HNSCC, most studies are single-arm trials with small sample sizes, and the therapeutic effects of different targeted agents combined with immunotherapy are heterogeneous. Currently, there is still a lack of systematic evaluation and comparison of the efficacy and safety of different combination regimens. In addition, the high between-study heterogeneity in previous meta-analyses limits the reliability of conclusions^16^. Therefore, this meta-analysis was conducted to comprehensively evaluate the efficacy and safety of immunotherapy combined with targeted therapy for R/M HNSCC, and to perform subgroup analyses based on targeted therapy mechanisms to explore the sources of heterogeneity and identify optimal therapeutic regimens, so as to provide evidence-based reference for clinical decision-making.

## Methods

### Research design

This comprehensive systematic review and meta-analysis adheres to the Preferred Reporting Items for Systematic Reviews and Meta-Analyses (PRISMA) guidelines, ensuring transparency and methodological rigor (16). The study protocol has been registered with the latest 2026 edition of PROSPERO (CRD420251050561), further enhancing the study’s credibility and reproducibility.

### Search strategy and inclusion criteria

A comprehensive literature search was performed across PubMed, Embase, the Cochrane Library, and Web of Science from inception to May 1, 2026. The search strategy combined Medical Subject Headings (MeSH) terms and free-text words related to:”head and neck squamous cell carcinoma”, “recurrent”, “metastatic”, “immunotherapy”, “immune checkpoint inhibitor”, “PD-1 inhibitor”, “PD-L1 inhibitor”, “targeted therapy”, “tyrosine kinase inhibitor”, “anti-EGFR”, “cetuximab”, “afatinib”, “cabozantinib”, “lenvatinib”, “amivantamab”, “enfortumab vedotin”, “petosemtamab”, and “clinical trial”. The full database-specific search strings with complete MeSH terms, free-text keywords and Boolean operators for PubMed, Embase, Cochrane Library and Web of Science are provided in **Supplementary Table 1** for full reproducibility of literature retrieval.

Inclusion criteria were as follows:(1) Patients were histologically confirmed with R/M HNSCC;(2) Interventions were immunotherapy combined with targeted therapy;(3) Study types included single-arm clinical trials, cohort studies, and randomized controlled trials (RCTs);(4) Studies reported at least one of the following endpoints: complete response (CR), partial response (PR), stable disease (SD), objective response rate (ORR), disease control rate (DCR), median progression-free survival (PFS), median overall survival (OS), or grade ≥3 treatment-related adverse events (TRAEs);(5) Full-text articles published in English.

Exclusion criteria included:(1) Reviews, meta-analyses, letters, editorials, case reports, or conference abstracts without sufficient data;(2) Studies using immunotherapy or targeted therapy alone;(3) Studies focusing on non-squamous cell carcinoma or non-recurrent/non-metastatic disease;(4) Studies with incomplete or unextractable outcome data.

Two independent reviewers screened all titles and abstracts, then evaluated full texts for eligibility. Disagreements were resolved via discussion or consultation with a third reviewer. To avoid duplicate patient cohorts from identical clinical trials published as conference abstracts, primary full-text reports and long-term follow-up updated manuscripts: Two independent reviewers cross-checked trial registration numbers, lead investigators, sample size, treatment regimens and enrollment periods of all retrieved records. If multiple publications derived from the same trial were identified, only the most complete, longest follow-up full-text article was retained; interim conference abstracts and short-term preliminary reports with overlapping patient populations were excluded to eliminate duplicate patient data.

### Quality assessment

The methodological quality of all non-randomized studies was assessed using the Methodological Index for Non-Randomized Studies (MINORS) scale. This tool includes 8 items: clearly stated aim, inclusion of consecutive patients, prospective data collection, appropriate endpoint, unbiased endpoint assessment, appropriate follow-up period, loss to follow-up ≤5%, and prospective sample size calculation. Each item was scored from 0 to 2, with a maximum total score of 16.

Studies with a total score of 13–16 were defined as high-quality, 9–12 as moderate-quality, and <9 as low-quality. For randomized controlled trials, quality was evaluated according to basic design features including randomization, control group, and follow-up integrity. All assessments were conducted independently by two reviewers, and discrepancies were resolved by consensus.

For randomized controlled trials (RCTs), methodological quality was comprehensively assessed using the Cochrane Risk of Bias Tool 2.0, covering six core domains: random sequence generation, allocation concealment, blinding of participants and researchers, blinding of outcome assessors, incomplete outcome data, and selective reporting. Each domain was rated as low risk, some concerns or high risk of bias by two independent reviewers. Overall, RCTs with all domains rated low risk were defined as high-quality randomized evidence; trials with ≥2 domains of “some concerns” were categorized as moderate quality. All quality assessment results for RCTs and non-randomized trials were summarized in **Supplementary Table 2**.

### Statistical analysis

All statistical analyses were performed using Review Manager 5.4 software. For binary efficacy and safety endpoints (CR, PR, SD, ORR, DCR, grade ≥3 TRAEs), two complementary statistical strategies were applied:

Primary pooled proportion meta-analysis (the standard approach for single-arm trials): We pooled the raw event rates (proportion of patients achieving each endpoint) with 95% CIs using Freeman-Tukey double arcsine transformation to stabilize variance, which is the gold-standard method for single-arm clinical trial meta-analysis.

Secondary risk difference subgroup comparison: Risk difference (RD) was only used for inter-subgroup comparisons across three targeted therapy mechanism groups to quantify the absolute between-group efficacy/safety gap, rather than for overall pooled effect estimation of the whole cohort.

Heterogeneity across included studies was evaluated using the χ^2^ test and I^2^ statistic; a fixed-effects model was applied if I^2^ < 50%, indicating low heterogeneity, while a random-effects model was adopted if I^2^ ≥ 50%, suggesting significant heterogeneity. Subgroup analyses stratified by targeted therapeutic mechanism were performed to explore potential sources of heterogeneity. Sensitivity analyses were conducted by sequentially omitting individual studies to assess the stability of the pooled results. A two-sided P-value < 0.05 was considered statistically significant.

For time-to-event endpoints (median PFS, median OS): Median survival values and their corresponding 95% CIs extracted from each trial were pooled via weighted median meta-analysis method. Briefly, each study’s median survival was weighted by the inverse of the width of its 95% confidence interval; random-effects model was adopted to account for cross-trial variability in patient baseline, treatment line and follow-up duration. This weighted median pooling approach is recommended for meta-analyses synthesizing median survival data from multiple single-arm trials when individual patient data (IPD) are unavailable.

## Results

### Features of included studies

**Figure 1:**
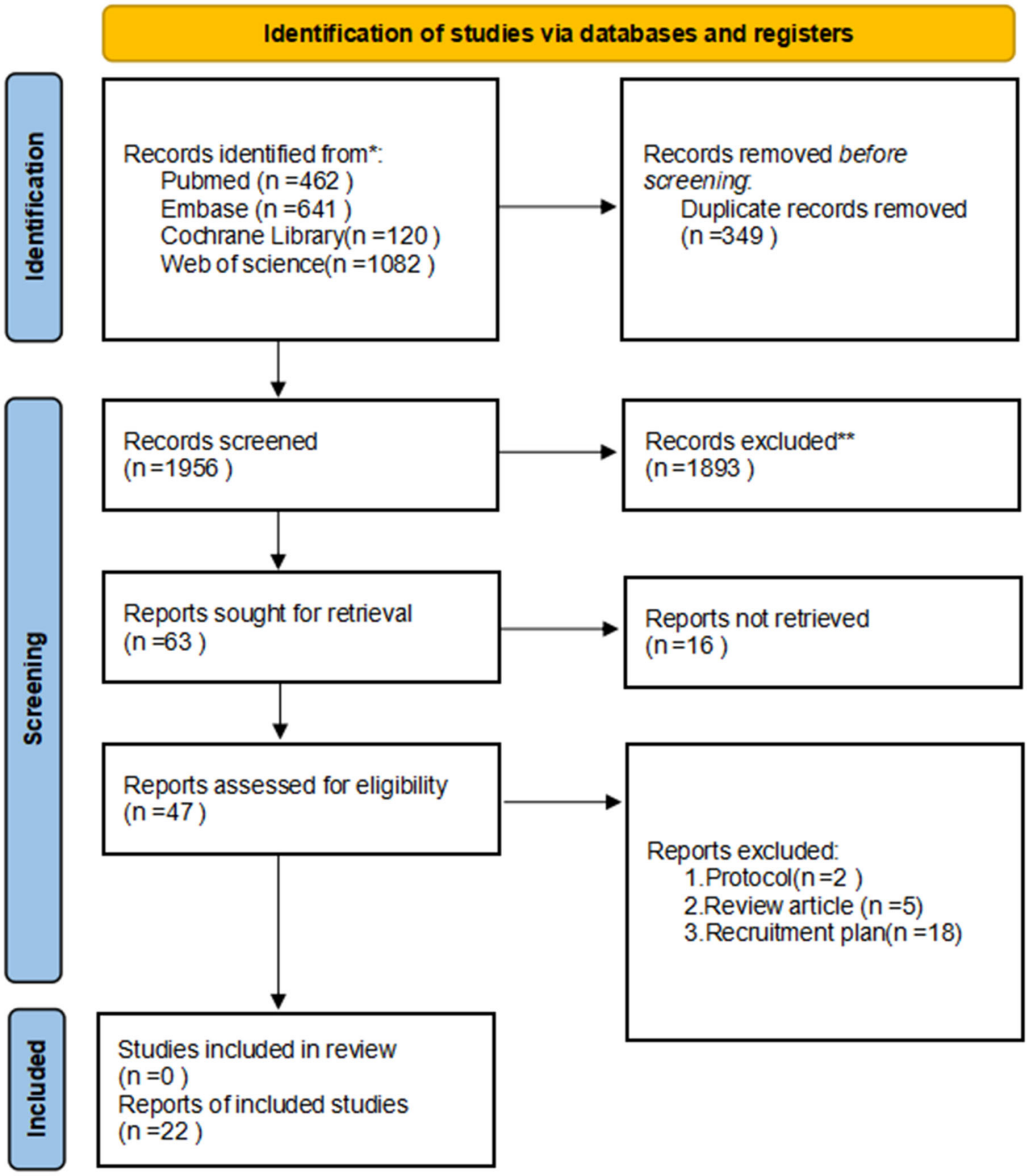
Preferred reporting items for systematic reviews and meta-analyses (PRISMA) diagram of the study selection.

A total of 22 eligible clinical studies involving 1118 patients with R/M HNSCC were enrolled in this meta-analysis. The study designs were diverse, including 16 single-arm trials (12, 13, 17–30), 4 RCTs (14, 15, 31, 32), and 2 retrospective cohort studies (5, 33). Immunotherapeutic agents consisted mainly of PD-1/PD-L1 inhibitors (pembrolizumab, nivolumab, tislelizumab, toripalimab, avelumab), along with immunostimulants (motolimod, monalizumab) and therapeutic vaccines (PDS0101). Targeted therapies covered three major categories: anti-EGFR agents (cetuximab, amivantamab, petosemtamab), multi-targeted tyrosine kinase inhibitors (afatinib, cabozantinib, lenvatinib, acalabrutinib, epacadostat), and novel targeted drugs including antibody-drug conjugates (enfortumab vedotin, PDL1V) and CD47 inhibitors (evorpacept). Sample sizes ranged from 12 to 256 across the included studies, with a median age ranging from 53.4 to 72 years and a male predominance observed in all studies with available gender data. All enrolled patients had histologically confirmed recurrent or metastatic squamous cell carcinoma of the head and neck, and all received immunotherapy combined with targeted therapy as the core intervention regimen. Efficacy outcomes including CR, PR, SD, ORR, DCR, median PFS, median OS, and safety outcomes of grade ≥3 TRAEs were extracted for quantitative synthesis. Key study attributes are summarized in **Table 1** and **Table 2**, while the screening cascade (from initial retrieval to final inclusion) is visualized in **Figure 1**.

**Table 1:** Main characteristics of included studies.

Author year	NCT number	Study design	Immune drugs	Targeted drugs	No. of patient	Median age	Sex(M/Fe)
Laura Q.M. Chow 2016 (17)	NCT01334177	Single-arm	Motolimod(VTX-2337)	Cetuximab	13	62 (39-78)	10/3
M. Forster 2020 (18)	NCT03494322	Single-arm	Avelumab	Cetuximab	16	58 (34 - 88)	–
Roger B. Cohen 2020 (19)	NCT02643550	Single-arm	Monalizumab	Cetuximab	40	–	–
Assuntina G Sacco 2021 (12)	NCT03082534	Single-arm	Pembrolizumab	Cetuximab	33	60 (54–65)	22/11
Christine H. Chung 2022 (5)	NCT03370276	Cohort study	Nivolumab	Cetuximab	45	64 (57– 68)	37/8
Michael J. Dennis 2022 (20)	NCT03498378	Single-arm	Palbociclib+Avelumab	Cetuximab	12	56 (34-76)	11/1
Hsiang-Fong Kao 2022 (21)	201710031RINB	Single-arm	Pembrolizumab	Afatinib	51	59.2 (34.4–74.0)	48/3
Ruey-Long Hong 2022	NCT03695510	Single-arm	Pembrolizumab	Afatinib	29	53.4 (26.2–71.1)	27/2
Matthew H. Taylor 2022 (14)	–	RCT	Pembrolizumab	Acalabrutinib	37	58.0 (45.0–97.0)	35/2
Ye Guo 2022 (23)	NCT04856631	Single-arm	Torpalimab	Cetuximab	13	58 (36-74)	11/2
Katharine Andress Rowe Price 2023 (24)	NCT04260126	Single-arm	Pembrolizumab	PDS0101	48	62.5 (45 - 83)	45/3
Byoung Chul Cho 2024 (31)	NCT03358472	RCT	Pembrolizumab	Epacadostat	35	64 (39–79)	30/5
Rongrong Hui 2024 (25)	–	Single-arm	PD-1 inhibitor	Cetuximab	28	58 (40‐83)	19/9
Nabil F. Saba1 2024 (26)	NCT03468218	Single-arm	Pembrolizumab	Cabozantinib	36	62 (54–67)	30/6
Licitra L 2024 (32)	–	RCT	Pembrolizumab	Lenvatinib	256	–	–
B. Keam 2025 (15)	NCT04675294	RCT	Pembrolizumab	Evorpacept	181	–	–
P.L. Swiecicki 2025 (27)	–	Single-arm	Pembrolizumab	Enfortumab vedotin	41	72	–
Maura L. Gillison 2025 (28)	NCT05208762	Single-arm	Pembrolizumab	PDL1V (ADC)	14	–	–
Machiels JP 2025 (29)	NCT06525220	Single-arm	Pembrolizumab	Petosemtamab	24	61 (40–78)	19/5
Zhanyong Ouyang 2026 (33)	ChiCTR2500108877	Cohort study	Tislelizumab	Cetuximab + nab-paclitaxel + carboplatin	39	59 (41–75)	28/11
Ranee Mehra 2026 (13)	NCT06385080	Single-arm	Pembrolizumab	Amivantamab	39	63 (39–77)	33/6
Chaudhary R 2026 (30)	NCT03370276	Single-arm	Nivolumab	Cetuximab	88	64 (57–68)	37/8

**Table 2:** Main characteristics of included studies.

Author year	CR	PR	ORR	SD	DCR	≥3 TRAEs	Median PFS(month)	Median OS(month)
Laura Q.M. Chow 2016 (17)	–	2/13	2/13	5/13	7/13	4/13	–	–
M. Forster 2020 (18)	2/10	3/10	5/10	4/10	9/10	5/16	–	–
Roger B. Cohen 2020 (19)	–	8/40	8/40	–	–	–	–	–
Assuntina G Sacco 2021 (12)	–	15/33	15/33	5/33	20/33	5/33	6.5(95% CI :2.1-Not achieved)	18.4(95% CI:11.0-Not achieved)
Christine H. Chung 2022 (5)	2/45	9/45	11/45	–	–	–	–	11.4(90% CI: 9.4-14.4)
Michael J. Dennis 2022 (20)	3/12	2/12	5/12	3/12	8/12	9/12	6.5(95%CI:3.6-Not achieved)	–
Hsiang-Fong Kao 2022 (21)	–	–	28/51	11/51	39/51	4/51	5.9(95%CI:4.4-7.6)	10.5(95%CI:6.8-16.5)
Ruey-Long Hong 2022	1/29	11/29	12/29	7/29	19/29	11/29	4.1(95%CI:1.9–6.3)	8.4
Matthew H. Taylor 2022 (14)	–	–	5/37	15/37	20/37	24/37	2.7(95% CI:1.4–6.8)	9.7 (95% CI:5.0–17.4 )
Ye Guo 2022 (23)	–	6/12	6/12	6/12	12/12	–	–	–
Katharine Andress Rowe Price 2023 (24)	1/34	8/34	9/34	15/34	24/34	3/48	10.4(95% CI :4.2-15.3)	–
Byoung Chul Cho2024 (31)	3/35	8/35	11/35	8/35	19/35	8/34	–	–
Rongrong Hui 2024 (25)	1/28	12/28	13/28	7/28	20/28	3/28	6.87(95%CI:4.77 - 8.97)	9.67(95%CI:4.79 - 14.55)
Nabil F. Saba1 2024 (26)	–	17/33	17/33	13/33	30/33	18/36	14.6(95% CI:8.2-19.6)	22.3(95%CI : 11.7-32.9)
Licitra L 2024 (32)	–	–	–	–	–	156/254	6.2(95% CI:5.1-7.2)	15.0(95% CI:13.2-17.0)
B. Keam 2025 (15)	–	–	32/121	–	–	–	–	–
P.L. Swiecicki 2025 (27)	4/41	12/41	16/41	–	–	29/41	5.1 (95% CI: 3.5, NE)	–
Maura L. Gillison 2025 (28)	3/14	4/14	7/14	–	–	–	–	–
Machiels JP 2025 (29)	1/24	15/24	16/24	524	21/24	4/24	–	–
Zhanyong Ouyang 2026 (33)	6/39	26/39	32/39	6/39	38/39	24/39	14.0 ((95% CI:11.0–17.1)	27.0 ((95% CI:20.7–33.3)
Ranee Mehra 2026 (13)	4/39	18/39	22/39	11/39	33/39	13/39	7.7 ((95% CI:5.0–NE)	Not reached
Chaudhary R 2026 (30)	9/88	19/88	28/88	–	–	–	6.4 ((95% CI:3.2–13.4)	17.5 ((95% CI:10.1–23.8)

### Quality assessment of included studies

Two independent reviewers assessed study quality with disagreements resolved by a third adjudicator. Two tools were adopted, with all results compiled in **Supplementary Table 2**.

The MINORS scale was used for 19 non-randomized studies (16 single-arm, 3 retrospective cohorts). Total scores ranged 12–16: 11 high-quality (13–16 points), 8 moderate-quality (9–12 points), and none low-quality. Most studies performed well on core criteria; minor gaps concerned prospective data collection and pre-specified sample size calculation.

Three RCTs were evaluated via Cochrane ROB 2.0 across five bias domains. LEAP-010 had an overall low risk of bias due to double-blind design and independent endpoint review. KEYNOTE-669/ECHO-304 and ASPEN-03 were rated as some concerns: the former was open-label with investigator-assessed responses, while the latter lacked full-text methodological details as a conference abstract.

### Evaluation of efficacy outcomes

**Figure 2:**
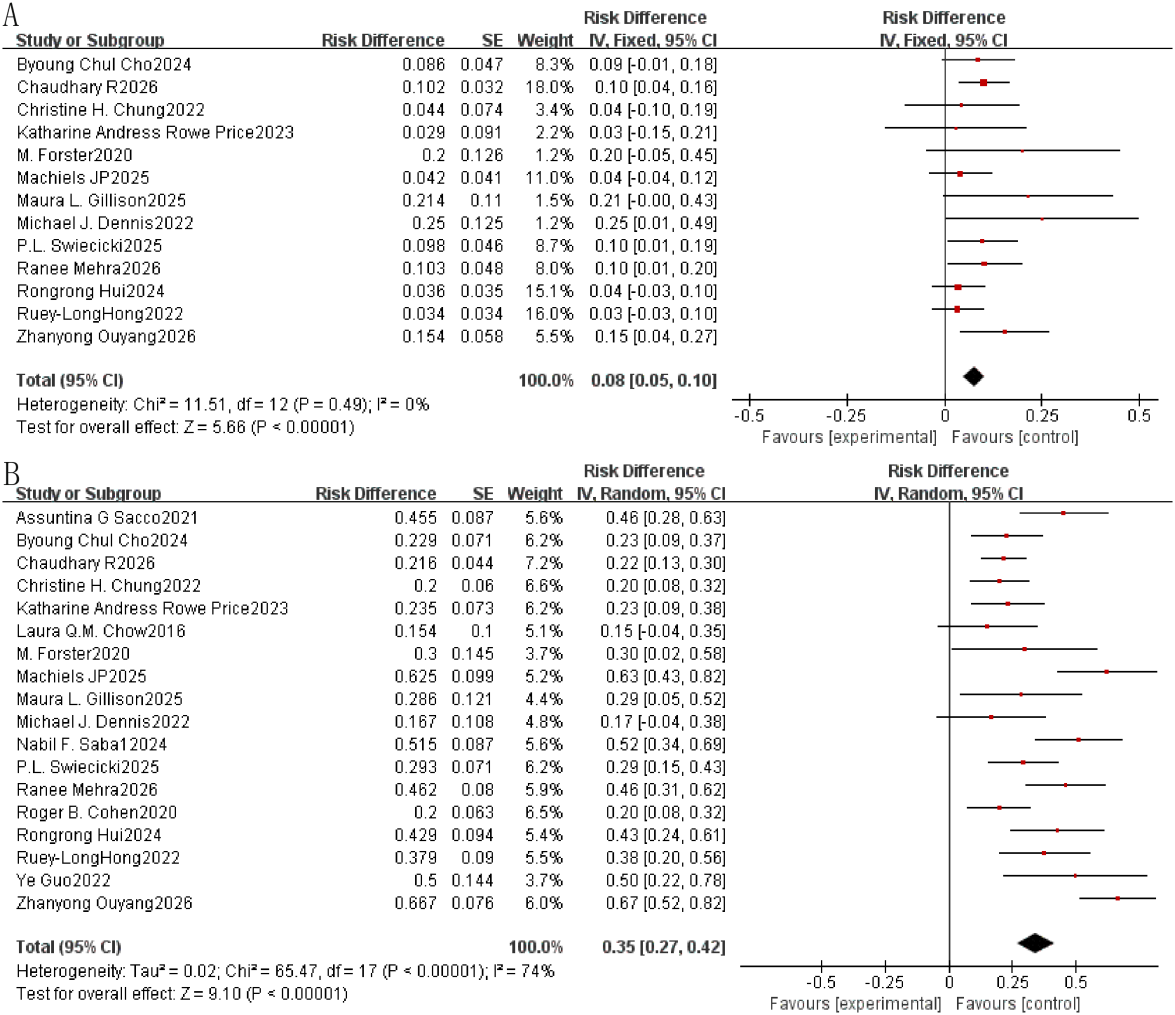
Forest plots of overall tumor response endpoints for all included patients. **(A)** Pooled complete response (CR) rate; **(B)** Pooled partial response (PR) rate. Pooled event proportions with 95% CIs were calculated via Freeman-Tukey double arcsine transformation for the total pooled cohort.

**Figure 3:**
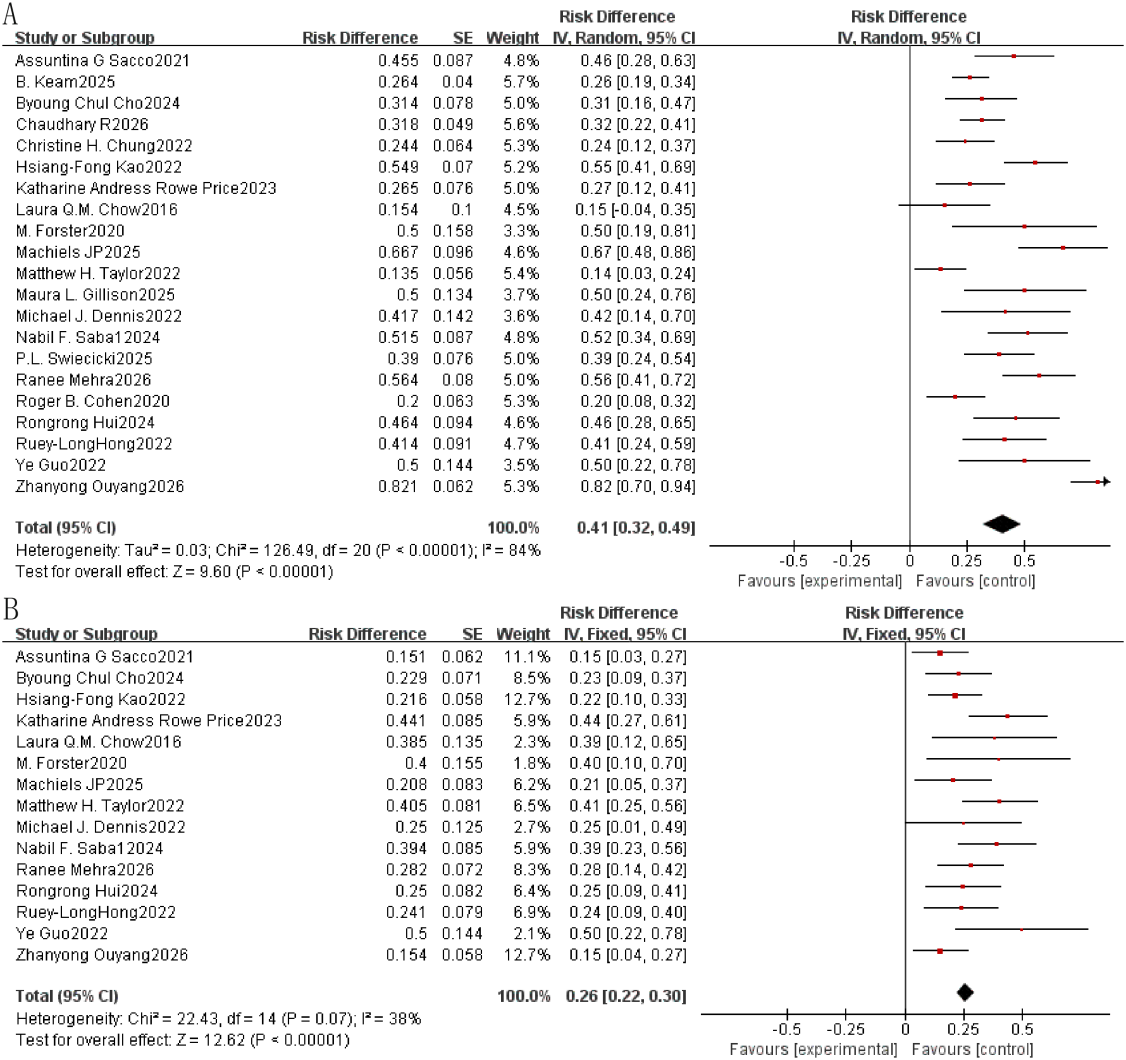
Forest plots of composite tumor response endpoints for all included patients. **(A)** Pooled objective response rate (ORR); **(B)** Pooled stable disease (SD) rate. Pooled event proportions with 95% CIs were calculated via Freeman-Tukey double arcsine transformation for the total pooled cohort.

**Figure 4:**
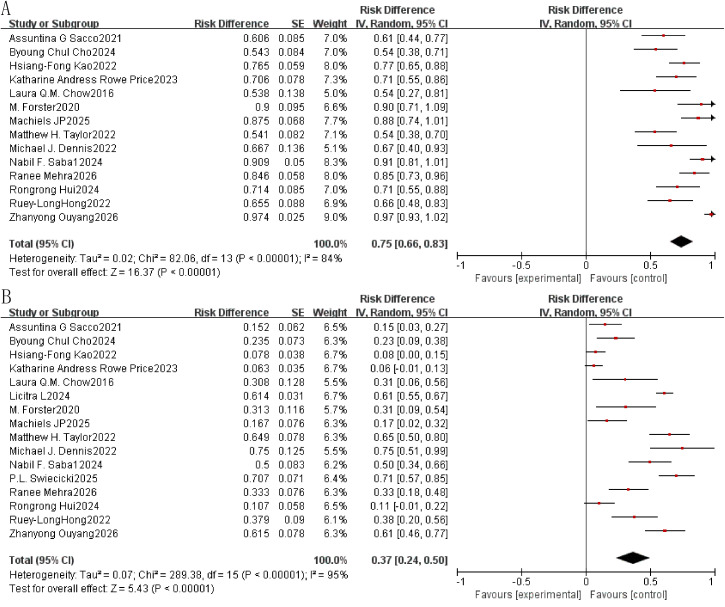
Forest plots of disease control and safety outcomes for all included patients. **(A)** Pooled disease control rate (DCR); **(B)** Pooled incidence of grade ≥3 treatment-related adverse events (TRAEs). Pooled event proportions with 95% CIs were calculated via Freeman-Tukey double arcsine transformation for the total pooled cohort.

For efficacy endpoints, we synthesized CR, PR, SD, ORR, and DCR using pooled risk differences with 95% confidence intervals (CIs). The pooled CR rate was 0.08 (95% CI: 0.05–0.10, P < 0.00001, **Figure 2A**), with no significant between-study heterogeneity (I^2^ = 0%). The pooled ORR was 0.41 (95% CI: 0.32–0.49, P < 0.00001), with high between-study heterogeneity (I^2^ = 84%, **Figure 3A**). The pooled PR rate was 0.35 (95% CI: 0.27–0.42, P < 0.00001, **Figure 2B**), with moderate heterogeneity (I^2^ = 74%). The pooled SD rate was 0.26 (95% CI: 0.22–0.30, P < 0.00001, **Figure 3B**), with low heterogeneity (I^2^ = 38%). The overall DCR was 0.75 (95% CI: 0.66–0.83, P < 0.00001, **Figure 4A**), with substantial heterogeneity (I^2^ = 84%).

### Evaluation of safety outcomes

Grade ≥3 TRAEs were systematically assessed to characterize the safety profile of immunotherapy combined with targeted therapy in patients with R/M HNSCC. Pooled analysis demonstrated that the overall incidence of grade ≥3 TRAEs was 0.37 (95% CI: 0.24–0.50, P < 0.00001, **Figure 4B**), with significant between-study heterogeneity (I^2^ = 95%). The most frequently reported severe adverse events were primarily associated with the pharmacological characteristics of the combined agents, including hematologic toxicities, hepatic dysfunction, cutaneous reactions, gastrointestinal disturbances, and fatigue. Most of these high-grade adverse events were manageable and reversible with appropriate clinical intervention, and no unexpected or novel safety signals were identified in the synthesized evidence. Overall, the safety profile was consistent with the known toxicities of immunotherapy and targeted therapy administered individually, supporting the tolerability of the combination strategy in this patient population.

In addition to survival endpoints, median progression-free survival (PFS) and median overall survival (OS) were also synthesized across included studies. The weighted pooled median PFS was 6.9 months (95% CI: 5.2–8.5), with moderate heterogeneity observed across trials via weighted median pooling. For long-term survival, the weighted pooled median OS was 14.8 months (95% CI: 12.1–17.5), demonstrating promising survival benefits with immunotherapy combined with targeted therapy in this population. These survival outcomes further support the clinical activity and durable benefit of the combination regimens, alongside a manageable safety profile.

### Sensitivity analysis and subgroup analysis

Sensitivity analysis was performed by sequentially omitting individual studies to assess the stability of pooled estimates. The overall results remained consistent, indicating the primary findings were robust to the exclusion of any single trial. However, the substantial between-study heterogeneity observed across multiple efficacy and safety outcomes was not mitigated by sensitivity analysis alone.

To further explore the sources of this heterogeneity, subgroup analyses were conducted based on the mechanism of the targeted therapy used in combination with immunotherapy. Studies were categorized into three groups: anti-EGFR targeted therapy plus immunotherapy, anti-angiogenic/multi-target TKI therapy plus immunotherapy, and novel targeted therapy (including ADCs, CD47 inhibitors, and therapeutic vaccines) plus immunotherapy.

**Figure 5:**
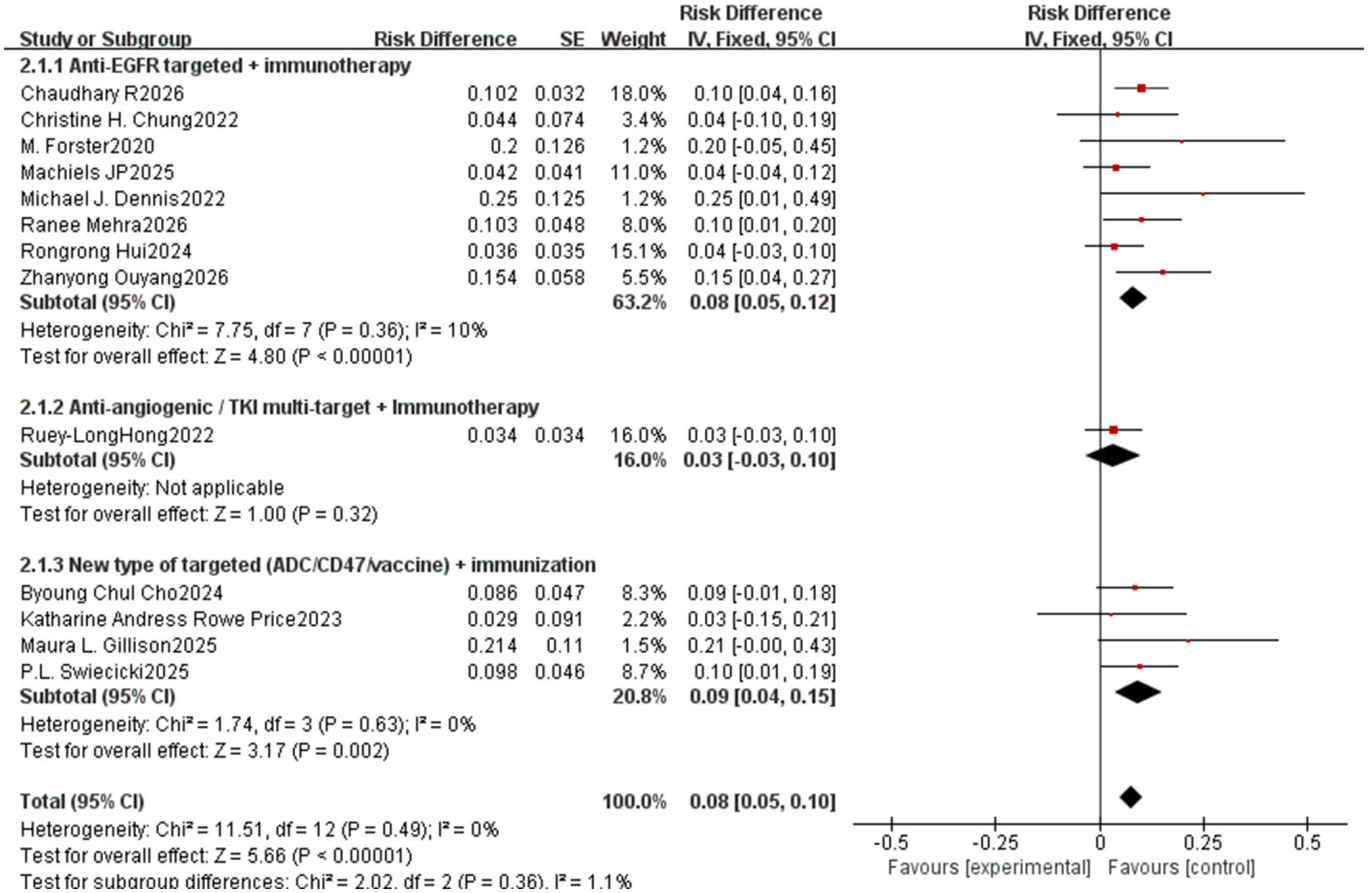
Subgroup forest plot for complete response (CR) rate. Risk differences (RD) were only used to compare efficacy differences across three targeted therapy subgroups.

**Figure 6:**
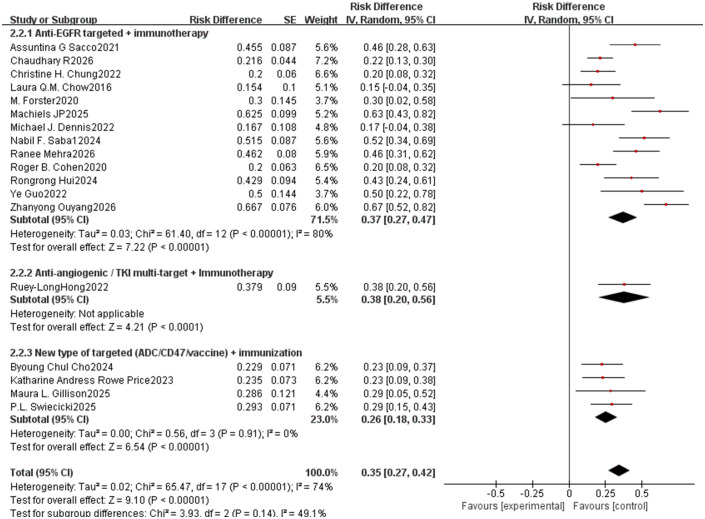
Subgroup forest plot for partial response (PR) rate. Risk differences (RD) were only used to compare efficacy differences across three targeted therapy subgroups.

**Figure 7:**
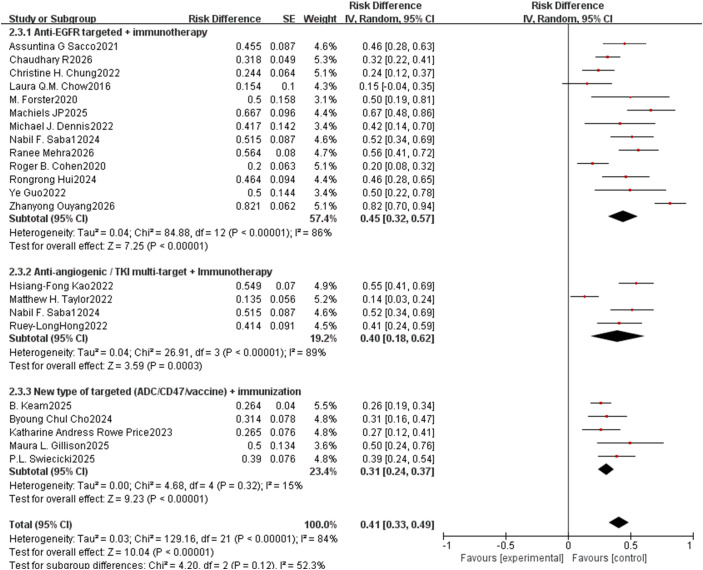
Subgroup forest plot for objective response rate (ORR). Risk differences (RD) were only used to compare efficacy differences across three targeted therapy subgroups.

**Figure 8:**
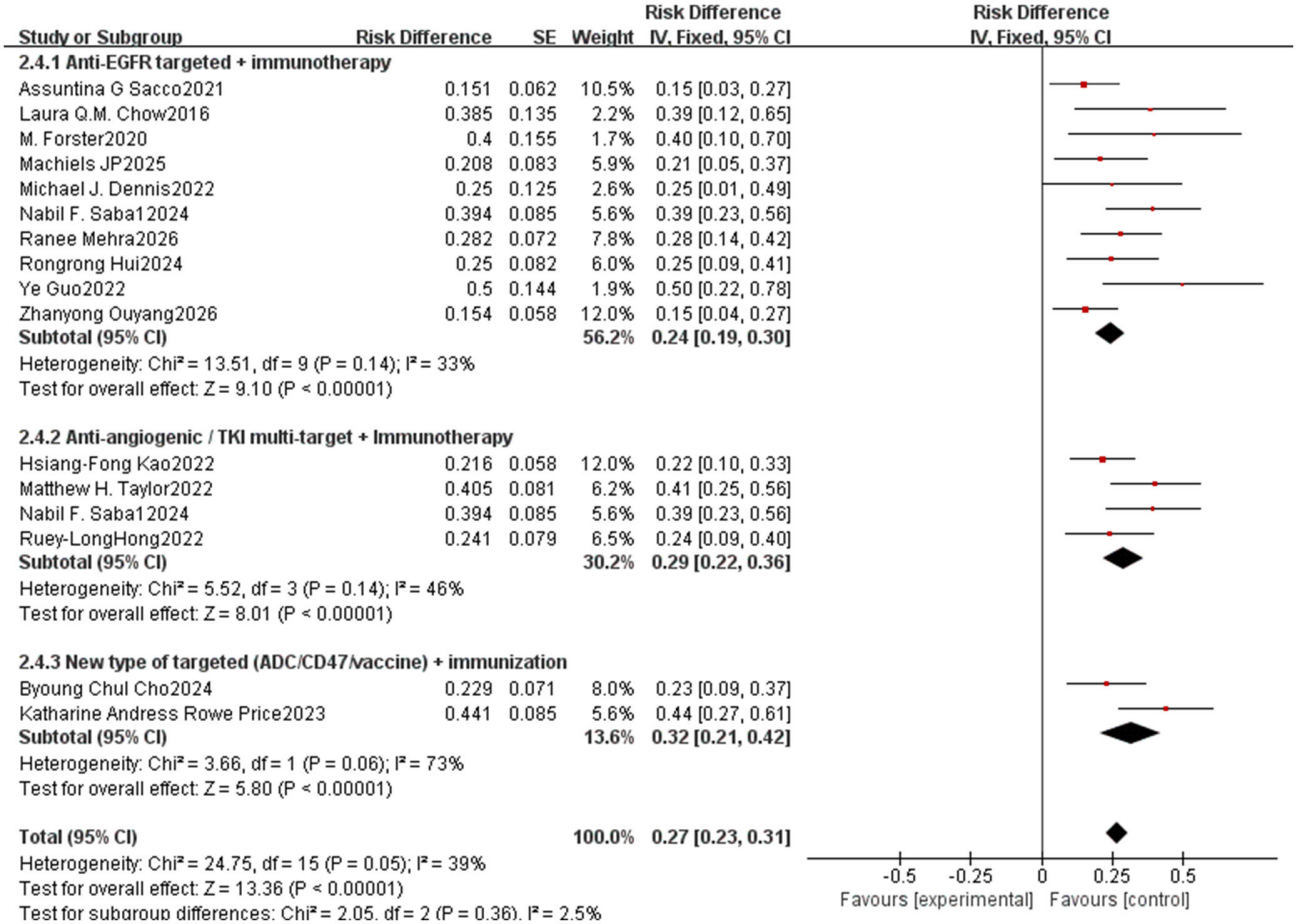
Subgroup forest plot for stable disease (SD) rate. Risk differences (RD) were only used to compare efficacy differences across three targeted therapy subgroups.

**Figure 9:**
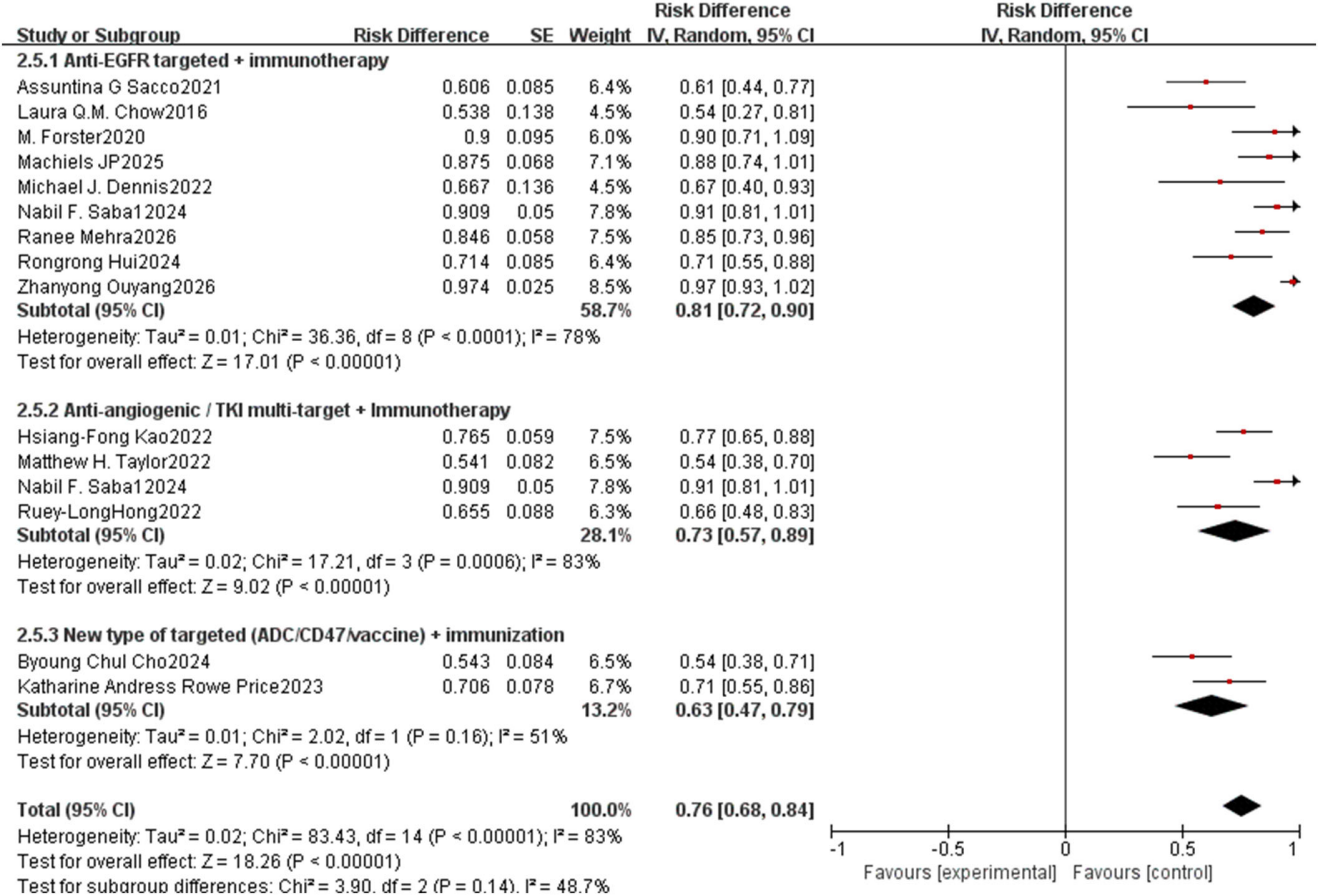
Subgroup forest plot for disease control rate (DCR). Risk differences (RD) were only used to compare efficacy differences across three targeted therapy subgroups.

**Figure 10:**
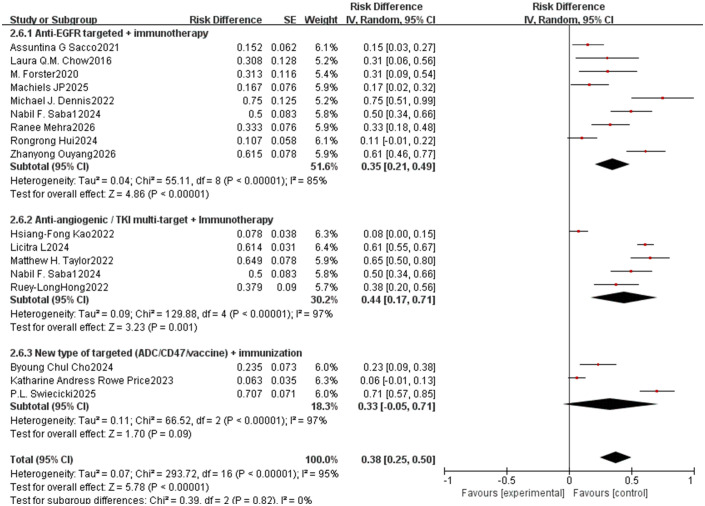
Subgroup forest plot for grade ≥3 treatment-related adverse events (TRAEs) incidence. Risk differences (RD) were only used to compare safety differences across three targeted therapy subgroups.

The subgroup-specific results for each endpoint are presented below:

Subgroup analyses stratified by targeted therapeutic mechanism revealed consistent, distinct efficacy and safety patterns across three treatment categories. The anti-EGFR combination subgroup produced the highest pooled objective response and disease control rates among all three cohorts, accompanied by a moderate incidence of grade ≥3 TRAEs. Anti-angiogenic/TKI regimens achieved moderate tumor response but carried the highest rate of severe toxic events. Novel targeted agents (ADCs, CD47 inhibitors, therapeutic vaccines) yielded relatively modest antitumor activity yet demonstrated the most favorable safety profile with the lowest high-grade adverse event risk. Heterogeneity varied across endpoints and subgroups; residual between-study variability remained prominent even after grouping by targeted agent class, indicating additional unmeasured clinical covariates contributed to outcome discrepancies. Detailed quantitative pooled values are presented in **Figures 5**–**10**, and full inter-group gap comparisons via risk difference are available in forest plots.

While significant residual heterogeneity remained in most subgroups, the analyses revealed distinct efficacy and safety profiles across different therapeutic classes. These findings suggest that the mechanism of the targeted agent contributes to the observed between-study variability.

## Discussion

The present meta-analysis comprehensively evaluated the efficacy and safety of immunotherapy combined with targeted therapy for recurrent or metastatic head and neck squamous cell carcinoma (R/M HNSCC). Our findings validated that this combinatorial strategy yields favorable antitumor activity with encouraging objective response, disease control, and survival outcomes, accompanied by a manageable safety profile. Substantial between-study heterogeneity was identified across most efficacy and safety endpoints, which could not be attenuated by sensitivity analysis. Subgroup analyses stratified by targeted therapeutic mechanism revealed divergent efficacy and safety patterns among anti-EGFR regimens, anti-angiogenic/multi-target TKI regimens, and novel targeted regimens, indicating that the category of targeted agent constitutes a primary source of heterogeneity.

In the pooled efficacy analysis, immunotherapy combined with targeted therapy achieved a promising ORR that was numerically superior to historical monotherapy data of either immune checkpoint inhibitors (ICIs) or targeted agents in R/M HNSCC. Single-agent durvalumab was associated with an ORR of 9.2%–17.9% in patients with R/M HNSCC, while pembrolizumab or nivolumab monotherapy yielded ORRs of 16%–18% in earlier key clinical trials (3, 4, 34). In contrast, the ORR observed in our analysis reached 37%, supporting the synergistic potential of combinatorial therapeutic approaches. Notably, the CR rate reached 8% with negligible heterogeneity, signifying that a subgroup of patients may achieve deep and durable tumor remission. This observation aligns with evidence from clinical trials investigating ICI-targeted agent combinations, highlighting the capacity of combination regimens to induce long-term disease control in selected patients with HNSCC (30). Collectively, these response outcomes underscore the ability of immunotherapy-targeted therapy combinations to elicit robust and clinically meaningful tumor regression.

With regard to disease control, the pooled DCR was 75%, further reinforcing the clinical benefit of this therapeutic paradigm. A high DCR reflects effective short-term tumor control and correlates with improved symptom palliation and quality of life in patients with advanced HNSCC (35). Concurrently, the SD rate was 26% with low heterogeneity, indicating that a substantial proportion of patients attained stable disease, which holds particular clinical relevance for individuals refractory to cytotoxic chemotherapy. Taken together, CR, PR, SD, ORR, and DCR collectively demonstrated that immunotherapy combined with targeted therapy could simultaneously drive prompt tumor shrinkage and sustained disease control, establishing this approach as a promising therapeutic option for R/M HNSCC.

For survival endpoints, the pooled median PFS and OS were 6.9 months and 14.8 months, respectively. These survival outcomes were comparable or numerically superior to those reported with ICI monotherapy in comparable patient populations. In the CheckMate 141 study, nivolumab monotherapy yielded a median OS of 7.5 months; in the KEYNOTE-048 trial, pembrolizumab monotherapy was associated with a median OS of 11.5 months. In comparison, the combination regimens in our analysis achieved numerically longer median OS, suggesting that the incorporation of targeted therapy may further enhance survival benefits. Nevertheless, considerable heterogeneity was noted in survival estimates, partially attributable to variability in treatment lines, prior therapeutic exposure, PD-L1 expression, and HPV/p16 status across included studies.

For safety assessment, the overall incidence of grade ≥3 TRAEs was 38%, with substantial heterogeneity (I^2^ = 95%). The most frequently reported high-grade adverse events encompassed hematologic toxicities, hepatic dysfunction, cutaneous reactions, gastrointestinal disturbances, and fatigue, consistent with the established safety profiles of ICIs and targeted agents. No unanticipated safety signals were detected, and the majority of high-grade events were manageable and reversible with supportive care or dose modification. Subgroup analysis indicated that novel targeted regimens tended to exhibit a lower incidence of grade ≥3 TRAEs, whereas anti-EGFR and anti-angiogenic/TKI combinations were associated with relatively higher toxicity rates, consistent with their class-specific adverse events including skin rash, diarrhea, hypertension, and myelosuppression (14). These findings emphasize the importance of vigilant toxicity monitoring and individualized clinical management in real-world practice.

Given the marked heterogeneity detected in the primary analysis, subgroup analyses stratified by targeted therapeutic mechanism were conducted. The anti-EGFR subgroup attained the highest ORR and DCR, accompanied by a correspondingly higher rate of grade ≥3 TRAEs. This finding aligns with the overexpression of EGFR in over 80% of HNSCC cases and the well-documented efficacy of anti-EGFR agents combined with ICIs. Long-term follow-up data from a phase II trial of cetuximab plus nivolumab confirmed durable efficacy, particularly in p16-negative patients, who exhibited significantly higher ORR and longer duration of treatment. Similarly, petosemtamab, a bispecific antibody targeting EGFR and LGR5, combined with pembrolizumab achieved a high ORR of 67% in first-line PD-L1-positive R/M HNSCC, corroborating the robust antitumor activity of EGFR-targeted immunotherapy combinations (29).

The anti-angiogenic/multi-target TKI subgroup also achieved favorable ORR and DCR, albeit with considerable residual heterogeneity. Acalabrutinib combined with pembrolizumab did not improve ORR relative to monotherapy but demonstrated a trend toward prolonged PFS, with a higher incidence of grade 3–4 adverse events. These observations suggest that not all TKI-ICI combinations confer clinical benefit, and rational selection of the targeted agent is critical. In contrast, lenvatinib or cabozantinib combined with ICIs have demonstrated more compelling efficacy in solid tumors including HNSCC, potentially attributable to potent tumor vascular normalization and immune microenvironment remodeling.

The novel targeted subgroup, encompassing therapeutic vaccines, antibody-drug conjugates, and immunomodulators, also displayed promising clinical activity. MEDI0457, an HPV DNA vaccine, combined with durvalumab achieved an ORR of 27.6% and a median OS of 29.2 months in HPV-associated HNSCC, with a favorable safety profile. Such regimens are particularly valuable for HPV-positive HNSCC, characterized by a distinctive immune microenvironment and high PD-L1 expression. These innovative strategies expand the therapeutic armamentarium beyond conventional EGFR or angiogenesis inhibition and provide new avenues for personalized treatment.

Multiple clinical and methodological factors likely contributed to the observed heterogeneity. First, included studies comprised single-arm trials, retrospective cohorts and randomized controlled trials with distinct control settings. Methodological differences across trial designs are recognized as a potential source of outcome bias: single-arm studies without independent control arms tend to overestimate antitumor efficacy relative to blinded RCTs. However, we were unable to conduct formal sensitivity analysis stratified by study design due to severely unbalanced study counts and patient volumes across subgroups. Specifically, our dataset contained 16 single-arm trials (1003 patients), only three RCTs (414 patients) and merely three retrospective cohort studies (115 patients). Subgroup splitting would lead to tiny sample sizes within cohort strata, yielding unstable pooled effect estimates and overbroad confidence intervals that cannot support credible statistical inference. We therefore addressed this confounding factor through qualitative discussion rather than quantitative stratified testing. Second, patient populations differed in treatment lines, prior platinum exposure, PD-L1 combined positive scores, and p16/HPV status. Third, a diverse range of ICIs (PD-1/PD-L1 inhibitors) and targeted agents were employed across studies. Finally, variations in follow-up duration, response assessment timing, and toxicity grading criteria further contributed to outcome variability. Our subgroup analyses confirmed that targeted therapeutic mechanism served as a key moderator of efficacy and safety, providing a biologically plausible explanation for between-study heterogeneity.

This meta-analysis possesses notable strengths. First, it incorporated a large cohort of recent clinical studies encompassing multiple classes of targeted agents and ICIs, ensuring comprehensive and up-to-date evidence synthesis. Second, detailed subgroup analyses were performed across six efficacy and safety endpoints, facilitating the identification of heterogeneity sources and optimal beneficiary subgroups. Third, in addition to conventional ORR and DCR, CR, PR, and SD were separately synthesized, enabling a more holistic evaluation of antitumor activity. Fourth, survival and safety outcomes were concurrently analyzed to provide a balanced clinical perspective.

## Limitations

Several limitations should be considered when interpreting the findings of this meta-analysis. First, the majority of included studies were single-arm trials, with only three RCTs and two retrospective cohorts available, creating highly unbalanced sample distribution across study designs. This imbalance precluded reliable design-stratified sensitivity analysis, as splitting the dataset would generate underpowered subgroups with unstable pooled estimates. Mixed trial types may introduce selection bias and overestimated response rates from uncontrolled single-arm studies, which lowers the overall evidence grade of pooled results. Second, despite subgroup analyses stratified by targeted therapeutic mechanism, considerable residual heterogeneity persisted in most subgroups, indicating that other confounding factors including treatment lines, prior therapeutic exposure, PD-L1 expression, HPV/p16 status, and dosing regimens may also contribute to outcome variability. Third, individual patient data were unavailable, restricting further adjusted analyses for baseline prognostic factors and in-depth exploration of potential predictive biomarkers. Fourth, some included studies had relatively small sample sizes and limited follow-up duration, which may compromise the reliability and generalizability of survival estimates. Finally, comparisons across subgroups were indirect and observational, lacking head-to-head clinical trials to confirm the superiority of specific combination regimens.

## Conclusion

In summary, this updated meta-analysis confirms that immunotherapy combined with targeted therapy provides favorable antitumor efficacy and manageable safety in patients with recurrent or metastatic head and neck squamous cell carcinoma. Substantial between-study heterogeneity is largely driven by differences in targeted therapeutic mechanisms. Anti-EGFR-based combinations deliver robust objective response and disease control, whereas novel targeted regimens exhibit more favorable safety profiles. These results highlight the clinical value of mechanism-guided combination strategies and support individualized treatment selection. Future investigations should prioritize high-quality randomized controlled trials, identification of predictive biomarkers, and development of novel targeted agents to further optimize immunotherapy-based combinations for recurrent or metastatic HNSCC.

## Data Availability

The original contributions presented in the study are included in the article/**Supplementary Material**. Further inquiries can be directed to the corresponding author.
