# Supplementary material for: Immunotherapy plus targeted therapy for recurrent or metastatic head and neck squamous cell carcinoma: an updated meta-analysis and subgroup analysis by target mechanism
Source: Front Oncol. 2026 Aug 3;16:1892431. doi: 10.3389/fonc.2026.1892431 (PMC13477942; doi:10.3389/fonc.2026.1892431)
Supplement: Supplementary file 1 [file Table1.docx]

| Author  year | NCT number | Study design | Immune drugs | Targeted drugs | No. of  patient | Median  age | Sex(M/Fe) |
| --- | --- | --- | --- | --- | --- | --- | --- |
| Laura Q.M. Chow2016 | NCT01334177 | Single-arm | Motolimod（VTX-2337） | Cetuximab | 13 | 62  (39-78) | 10/3 |
| M. Forster2020 | NCT03494322 | Single-arm | Avelumab | Cetuximab | 16 | 58  (34 - 88) | - |
| Roger B. Cohen2020 | NCT02643550 | Single-arm | Monalizumab | Cetuximab | 40 | - | - |
| Assuntina G Sacco2021 | NCT03082534 | Single-arm | Pembrolizumab | Cetuximab | 33 | 60  (54–65) | 22/11 |
| Christine H. Chung2022 | NCT03370276 | Cohort study | Nivolumab | Cetuximab | 45 | 64  (57– 68) | 37/8 |
| Michael J. Dennis2022 | NCT03498378 | Single-arm | Palbociclib+Avelumab | Cetuximab | 12 | 56  (34-76) | 11/1 |
| Hsiang‑Fong Kao2022 | 201710031RINB | Single-arm | Pembrolizumab | Afatinib | 51 | 59.2 (34.4–74.0) | 48/3 |
| Ruey-LongHong2022 | NCT03695510 | Single-arm | Pembrolizumab | Afatinib | 29 | 53.4  (26.2–71.1) | 27/2 |
| Matthew H. Taylor2022 | - | RCT | Pembrolizumab | Acalabrutinib | 37 | 58.0  (45.0–97.0) | 35/2 |
| Ye Guo2022 | NCT04856631 | Single-arm | Torpalimab | Cetuximab | 13 | 58  （36-74） | 11/2 |
| Katharine Andress Rowe Price2023 | NCT04260126 | Single-arm | Pembrolizumab | PDS0101 | 48 | 62.5  （45 - 83） | 45/3 |
| Byoung Chul Cho2024 | NCT03358472 | RCT | Pembrolizumab | Epacadostat | 35 | 64  (39–79) | 30/5 |
| Rongrong Hui2024 | - | Single-arm | PD-1 inhibitor | Cetuximab | 28 | 58  (40‐83) | 19/9 |
| Nabil F. Saba12024 | NCT03468218 | Single-arm | Pembrolizumab | Cabozantinib | 36 | 62  (54–67) | 30/6 |
| Licitra L2024 | - | RCT | Pembrolizumab | Lenvatinib | 256 | - | - |
| B. Keam2025 | NCT04675294 | RCT | Pembrolizumab | Evorpacept | 181 | - | - |
| P.L. Swiecicki2025 | – | Single-arm | Pembrolizumab | Enfortumab vedotin | 41 | 72 | - |
| Maura L. Gillison2025 | NCT05208762 | Single-arm | Pembrolizumab | PDL1V (ADC) | 14 | - | - |
| Machiels JP2025 | NCT06525220 | Single-arm | Pembrolizumab | Petosemtamab | 24 | 61 (40–78) | 19/5 |
| Zhanyong Ouyang2026 | ChiCTR2500108877 | Cohort study | Tislelizumab | Cetuximab + nab-paclitaxel + carboplatin | 39 | 59 (41–75) | 28/11 |
| Ranee Mehra2026 | NCT06385080 | Single-arm | Pembrolizumab | Amivantamab | 39 | 63 (39–77) | 33/6 |
| Chaudhary R2026 | NCT03370276 | Single-arm | Nivolumab | Cetuximab | 88 | 64 (57–68) | 37/8 |

TABLE 1 Main characteristics of included studies
