# Supplementary material for: Immunotherapy plus targeted therapy for recurrent or metastatic head and neck squamous cell carcinoma: an updated meta-analysis and subgroup analysis by target mechanism
Source: Front Oncol. 2026 Aug 3;16:1892431. doi: 10.3389/fonc.2026.1892431 (PMC13477942; doi:10.3389/fonc.2026.1892431)
Supplement: Supplementary file 2 [file Table2.docx]

| Author  year | CR | PR | ORR | SD | DCR | ≥3  TRAEs | Median PFS(month) | Median OS(month) |
| --- | --- | --- | --- | --- | --- | --- | --- | --- |
| Laura Q.M. Chow2016 | - | 2/13 | 2/13 | 5/13 | 7/13 | 4/13 | - | - |
| M. Forster2020 | 2/10 | 3/10 | 5/10 | 4/10 | 9/10 | 5/16 | - | - |
| Roger B. Cohen2020 | - | 8/40 | 8/40 | - | - | - | - | - |
| Assuntina G Sacco2021 | - | 15/33 | 15/33 | 5/33 | 20/33 | 5/33 | 6.5(95% CI :2.1-Not achieved） | 18.4(95% CI:11.0-Not achieved） |
| Christine H. Chung2022 | 2/45 | 9/45 | 11/45 | - | - | - | - | 11.4(90% CI: 9.4-14.4) |
| Michael J. Dennis2022 | 3/12 | 2/12 | 5/12 | 3/12 | 8/12 | 9/12 | 6.5(95%CI:3.6-Not achieved) | - |
| Hsiang‑Fong Kao2022 | - | - | 28/51 | 11/51 | 39/51 | 4/51 | 5.9(95%CI：4.4-7.6) | 10.5(95%CI:6.8-16.5) |
| Ruey-LongHong2022 | 1/29 | 11/29 | 12/29 | 7/29 | 19/29 | 11/29 | 4.1(95%CI:1.9–6.3) | 8.4 |
| Matthew H. Taylor2022 | - | - | 5/37 | 15/37 | 20/37 | 24/37 | 2.7(95% CI:1.4–6.8) | 9.7 (95% CI:5.0–17.4 ) |
| Ye Guo2022 | - | 6/12 | 6/12 | 6/12 | 12/12 | - | - | - |
| Katharine Andress Rowe Price2023 | 1/34 | 8/34 | 9/34 | 15/34 | 24/34 | 3/48 | 10.4(95% CI :4.2-15.3) | - |
| Byoung Chul Cho2024 | 3/35 | 8/35 | 11/35 | 8/35 | 19/35 | 8/34 | - | - |
| Rongrong Hui2024 | 1/28 | 12/28 | 13/28 | 7/28 | 20/28 | 3/28 | 6.87(95%CI:4.77 - 8.97) | 9.67(95%CI:4.79 - 14.55) |
| Nabil F. Saba12024 | - | 17/33 | 17/33 | 13/33 | 30/33 | 18/36 | 14.6(95% CI:8.2-19.6) | 22.3(95%CI : 11.7-32.9) |
| Licitra L2024 | - | - | - | - | - | 156/254 | 6.2(95% CI:5.1-7.2) | 15.0(95% CI:13.2-17.0) |
| B. Keam2025 | – | – | 32/121 | – | – | – | – | – |
| P.L. Swiecicki2025 | 4/41 | 12/41 | 16/41 | – | – | 29/41 | 5.1 (95% CI: 3.5, NE) | – |
| Maura L. Gillison2025 | 3/14 | 4/14 | 7/14 | – | – | – | – | – |
| Machiels JP2025 | 1/24 | 15/24 | 16/24 | 524 | 21/24 | 4/24 | – | – |
| Zhanyong Ouyang2026 | 6/39 | 26/39 | 32/39 | 6/39 | 38/39 | 24/39 | 14.0 ((95% CI:11.0–17.1) | 27.0 ((95% CI:20.7–33.3) |
| Ranee Mehra2026 | 4/39 | 18/39 | 22/39 | 11/39 | 33/39 | 13/39 | 7.7 ((95% CI:5.0–NE) | Not reached |
| Chaudhary R2026 | 9/88 | 19/88 | 28/88 | – | – | – | 6.4 ((95% CI:3.2–13.4) | 17.5 ((95% CI:10.1–23.8) |

TABLE 2 Main characteristics of included studies

CR:Complete response ; PR:Partial response ; SD:Stable disease ; ORR:Objective response rate ; DCR:Disease control rate ; TRAEs:Treatment-related adverse event ; PFS:Progression-Free Survival ; OS:Overall Survival
