# Supplementary material for: Immunotherapy plus targeted therapy for recurrent or metastatic head and neck squamous cell carcinoma: an updated meta-analysis and subgroup analysis by target mechanism
Source: Front Oncol. 2026 Aug 3;16:1892431. doi: 10.3389/fonc.2026.1892431 (PMC13477942; doi:10.3389/fonc.2026.1892431)
Supplement: Supplementary file 3 [file Table3.docx]

**PubMed**

(Immunotherapy OR Immunotherapies OR pembrolizumab OR toripalimab OR avelumab OR ipilimumab OR atezolizumab OR nivolumab OR PD-1 OR PD-L1) AND (Targeted Therapy, Molecular OR Therapy, Molecular Targeted OR Targeted Molecular Therapy OR Molecular Therapy, Targeted OR Targeted Molecular Therapies OR Therapy, Targeted) AND (EGFR OR epidermal growth factor receptor) AND (Head and neck squamous cell carcinoma OR HNSCC OR Carcinoma, Squamous Cell of Head and Neck OR Head and Neck OR Head and Neck Squamous Cell Carcinoma OR Oral Squamous Cell Carcinoma) AND (recurrent OR metastatic OR relapsed OR R/M) AND ("Clinical Trial"[Publication Type] OR "Clinical Trial, Phase I"[Publication Type] OR "Clinical Trial, Phase II"[Publication Type] OR "Clinical Trial, Phase III"[Publication Type] OR cohort study)

**Embase**

(Immunotherapy OR Immunotherapies OR pembrolizumab OR toripalimab OR avelumab OR ipilimumab OR atezolizumab OR nivolumab OR PD-1 OR PD-L1) AND (Targeted Therapy, Molecular OR Therapy, Molecular Targeted OR Targeted Molecular Therapy OR Molecular Therapy, Targeted OR Targeted Molecular Therapies OR Therapy, Targeted) AND (EGFR OR epidermal growth factor receptor) AND (Head and neck squamous cell carcinoma OR HNSCC OR Carcinoma, Squamous Cell of Head and Neck OR Head and Neck OR Head and Neck Squamous Cell Carcinoma OR Oral Squamous Cell Carcinoma) AND (recurrent OR metastatic OR relapsed OR R/M) AND (clinical trial OR phase 1 clinical trial OR phase 2 clinical trial OR phase 3 clinical trial OR cohort study) NOT (review NOT systematic review NOT meta analysis)

**Cochrane Library**

(Immunotherapy OR Immunotherapies OR pembrolizumab OR toripalimab OR avelumab OR ipilimumab OR atezolizumab OR nivolumab OR PD-1 OR PD-L1) AND (Targeted Therapy, Molecular OR Therapy, Molecular Targeted OR Targeted Molecular Therapy OR Molecular Therapy, Targeted OR Targeted Molecular Therapies OR Therapy, Targeted) AND (EGFR OR epidermal growth factor receptor) AND (Head and neck squamous cell carcinoma OR HNSCC OR Carcinoma, Squamous Cell of Head and Neck OR Head and Neck OR Head and Neck Squamous Cell Carcinoma OR Oral Squamous Cell Carcinoma) AND (recurrent OR metastatic OR relapsed OR R/M) AND (clinical trial OR cohort study)

**Web of Science**

TS=(Immunotherapy OR Immunotherapies OR pembrolizumab OR toripalimab OR avelumab OR ipilimumab OR atezolizumab OR nivolumab OR PD-1 OR PD-L1) AND TS=(Targeted Therapy, Molecular OR Therapy, Molecular Targeted OR Targeted Molecular Therapy OR Molecular Therapy, Targeted OR Targeted Molecular Therapies OR Therapy, Targeted) AND TS=(EGFR OR epidermal growth factor receptor) AND TS=(Head and neck squamous cell carcinoma OR HNSCC OR Carcinoma, Squamous Cell of Head and Neck OR Head and Neck OR Head and Neck Squamous Cell Carcinoma OR Oral Squamous Cell Carcinoma) AND TS=(recurrent OR metastatic OR relapsed OR R/M) AND TS=(clinical trial OR phase I trial OR phase II trial OR phase III trial OR cohort study) AND DT=Article AND LA=English
