# Supplementary material for: Immunotherapy plus targeted therapy for recurrent or metastatic head and neck squamous cell carcinoma: an updated meta-analysis and subgroup analysis by target mechanism
Source: Front Oncol. 2026 Aug 3;16:1892431. doi: 10.3389/fonc.2026.1892431 (PMC13477942; doi:10.3389/fonc.2026.1892431)
Supplement: Supplementary file 4 [file Table4.docx]

**Supplementary Table 2** Assessment of non-randomized controlled trials in the version of MINORS.

| Study | A clearly  stated  aim | Inclusion of  consecutive  patients | Prospective  collection of  data | Endpoint  appropriate  to the study  aim | Unbiased  assessment  of endpoints | Follow-up  period  appropriate  to the major  endpoint | Loss to  follow  up not  exceeding  5% | Prospective  calculation  of the study  size | Total score |
| --- | --- | --- | --- | --- | --- | --- | --- | --- | --- |
| Laura Q.M. Chow2016 | 2 | 2 | 2 | 1 | 1 | 1 | 2 | 1 | 12 |
| M. Forster2020 | 2 | 2 | 2 | 1 | 1 | 1 | 2 | 1 | 12 |
| Roger B. Cohen2020 | 2 | 2 | 2 | 1 | 2 | 1 | 2 | 2 | 14 |
| Assuntina G Sacco2021 | 2 | 2 | 2 | 2 | 2 | 2 | 2 | 2 | 16 |
| Christine H. Chung2022 | 2 | 2 | 2 | 1 | 2 | 2 | 2 | 1 | 14 |
| Michael J. Dennis2022 | 2 | 2 | 2 | 1 | 2 | 1 | 2 | 1 | 13 |
| Hsiang‑Fong Kao2022 | 2 | 2 | 2 | 2 | 2 | 2 | 2 | 2 | 16 |
| Ruey-LongHong2022 | 2 | 2 | 2 | 2 | 2 | 2 | 2 | 1 | 15 |
| Matthew H. Taylor2022 | 2 | 2 | 2 | 2 | 1 | 1 | 1 | 1 | 12 |
| Ye Guo2022 | 2 | 2 | 2 | 1 | 2 | 1 | 2 | 1 | 13 |
| Katharine Andress Rowe Price2023 | 2 | 2 | 2 | 1 | 2 | 1 | 2 | 1 | 13 |
| Rongrong Hui2024 | 2 | 2 | 2 | 1 | 1 | 1 | 2 | 1 | 12 |
| Nabil F. Saba12024 | 2 | 2 | 2 | 2 | 2 | 2 | 2 | 1 | 15 |
| P.L. Swiecicki2025 | 2 | 2 | 1 | 2 | 2 | 2 | 2 | 1 | 14 |
| Maura L. Gillison2025 | 2 | 2 | 1 | 2 | 1 | 2 | 2 | 1 | 13 |
| Machiels JP2025 | 2 | 2 | 2 | 2 | 2 | 2 | 2 | 1 | 15 |
| Zhanyong Ouyang2026 | 2 | 2 | 1 | 2 | 2 | 2 | 2 | 1 | 14 |
| Ranee Mehra2026 | 2 | 2 | 1 | 2 | 2 | 2 | 2 | 1 | 14 |
| Chaudhary R2026 | 2 | 2 | 2 | 2 | 2 | 2 | 2 | 1 | 15 |

**Summary Table of ROB 2.0 Assessment**

| **Study** | **Year** | **D1 Randomization Process** | **D2 Deviations from Intended Interventions** | **D3 Missing Outcome Data** | **D4 Measurement of the Outcome** | **D5 Selection of the Reported Result** | **Overall Risk of Bias** |
| --- | --- | --- | --- | --- | --- | --- | --- |
| KEYNOTE-669/ECHO-304 | 2024 | Low | Some concerns | Low | Some concerns | Low | **Some concerns** |
| LEAP-010 | 2024 | Low | Low | Low | Low | Low | **Low risk** |
| ASPEN-03 | 2025 | Some concerns | Some concerns | Some concerns | Some concerns | Some concerns | **Some concerns** |
